# Fifteen complete chloroplast genomes of *Trapa* species (Trapaceae): insight into genome structure, comparative analysis and phylogenetic relationships

**DOI:** 10.1186/s12870-022-03608-7

**Published:** 2022-05-05

**Authors:** Xiangrong Fan, Wuchao Wang, Godfrey K. Wagutu, Wei Li, Xiuling Li, Yuanyuan Chen

**Affiliations:** 1 Key Laboratory of Aquatic Botany and Watershed Ecology, Wuhan Botanical Garden, Chinese Academy of Sciences, Wuhan, 430074 People’s Republic of China; 2 College of Science, Tibet University, Lhasa, 850000 People’s Republic of China; 3 Research Center for Ecology and Environment of Qinghai-Tibetan Plateau, Tibet University, Lhasa, 850000 People’s Republic of China; 4 Center of Plant Ecology, Core Botanical Gardens, Chinese Academy of Sciences, Wuhan, 430074 People’s Republic of China; 5 College of Life Science, Linyi University, Linyi, 276000 People’s Republic of China

**Keywords:** *Trapa*, Complete chloroplast genomes, Comparative analysis, Species identification, Phylogeography

## Abstract

**Background:**

*Trapa* L. is a floating-leaved aquatic plant with important economic and ecological values. However, the species identification and phylogenetic relationship within *Trapa* are still controversial, which necessitates the need for plastid genome information of *Trapa*. In this study, complete chloroplast genomes of 13 *Trapa* species/taxa were sequenced and annotated. Combined with released sequences, comparative analyses of chloroplast genomes were performed on the 15 *Trapa* species/taxa for the first time.

**Results:**

The *Trapa* chloroplast genomes exhibited typical quadripartite structures with lengths from 155,453 to 155,559 bp. The gene orders and contents within *Trapa* were conservative, but several changes were found in the microstructure. The intron loss of *rpl2*, also detected in Lythraceae, was found in all *Trapa* species/taxa, suggesting close genetic relationship between Lythraceae and Trapaceae. Notably, two small-seed species (*T. incisa* and *T*. *maximowiczii*) showed the smallest genome size with 155,453 and 155,477 bp, respectively. Each cp genome contained the same 130 genes consisting of 85 protein-coding genes, 37 tRNA genes and 8 rRNA genes. *Trapa* species/taxa showed 37 (*T. incisa* and *T. maximowiczii*) to 41 (*T. sibirica*) long repeats, including forward, palindromic, reversed and complementary repeats. There were 110 (*T. quadrispinosa*) to 123 (*T. incisa* and *T. maximowiczii*) SSR (simple sequence repeat) loci in *Trapa* chloroplast genomes. Comparative analyses revealed that two hotspot regions (*atpA—atpF* and *rps*2*—rpoC*2) in *Trapa* chloroplast genomes could be served as potential molecular markers. Three phylogenetic analyses (ML, MP and BI) consistently showed that there were two clusters within *Trapa*, including large- and small-seed species/taxa, respectively; for the large-seed *Trapa*, they clustered according to their geographical origin and tubercle morphology on the surface of seeds.

**Conclusion:**

In summary, we have acquired the sequences of 13 *Trapa* chloroplast genomes, and performed the comparative analyses within *Trapa* for the first time. The results have helped us better identify the *Trapa* species/taxa and deepen the understanding of genetic basis and phylogenetic relationship of *Trapa*, which will facilitate the effective management and utilization of the important genetic resources in the future.

**Supplementary Information:**

The online version contains supplementary material available at 10.1186/s12870-022-03608-7.

## Background

Trapaceae, containing the only genus *Trapa*, is an annual floating-leaved aquatic herb naturally distributed in tropical, subtropical and temperate regions of Eurasia and Africa, and invading North America and Australia [1]. APG II (The Angiosperm Phylogeny Group) [2] equated Trapaceae with Lythraceae. However, a handful of morphological differences exist between the two families. For example, flowers of Trapaceae are solitary, 4-merous and actinomorphic, with half-inferior and slightly perigynous ovaries; Lythraceae has racemes or cymes, and the flowers are usually 4-, 6- or 8-merous, regular or irregular, with obvious perigynous ovaries. Therefore, Trapaceae is still be used today by some researchers [1]. *Trapa* has important edible value because of high content of starch in seeds, and it has been widely cultivated as an important aquatic crop in China and India [3]. *Trapa* seed pericarps were traditional herb medicines in China, and recent studies found that the extract of seed pericarps had bioactive components to restrain cancer, atherosclerosis, inflammation and oxidation [4–8]. Additionally, *Trapa* plants can be used to purify water bodies due to their excellent performance in absorbing heavy metals and nutrients [9, 10]. Therefore, *Trapa* has important economic and ecological values. However, many *Trapa* species are becoming endangered or even locally extinct due to human interferences in Europe and China [11] (Chen et al., field observations). Conversely, *Trapa* plants were notorious intruders in Canada and the northeastern United States.

The knowledge of species identification and phylogenetic relationship is essential to effectively managing genetic resources [12, 13]. However, the genus *Trapa* possesses complicated morphological variations, but lacks effective diagnostic criteria. Therefore, researchers have held sharp different opinions on taxonomic classification of *Trapa*, with 1 or 2 polymorphic species or more than 20, 30 or 70 species within the genus [1, 14–19]. The phylogenetic relationships of *Trapa* species are still unresolved despite the efforts put in pollen morphology, cytology, quantitative classification and ontogenesis [20–24]. For example, Xiong et al. [25, 26] proposed that *T. acornis* Nakano and *T. bispinosa* Roxb were closely related based on the results of quantitative classification; contrastingly, Ding et al. [21] found that *T. acornis* was closely related to *T. quadrispinosa* Roxb based on their pollen morphology. Quantitative classification studies showed that the *Trapa* species/taxa with seeds of similar size evolved closely [24, 27], which was proved by the results of allozymes [28]. Additionally, molecular methods further illustrated that the *Trapa* species with small seeds, *T. incisa* and *T. maximowiczii*, were the primitive species of the genus [29, 30]. For the large-seed *Trapa* species, a close relationship was found between *T. bispinosa* and *T. quadrispinosa* based on RAPDs and AFLPs [29–31]. It is worth noting that there are two diversity centers in *Trapa* genus, one in the mid-lower reaches of Yangtze River, and the other in the Tumen River and Amur River basins [32, 33]. However, most previous studies involved fewer species/taxa, and the sampling areas were confined to the mid-lower reaches of Yangtze River. Additionally, their experimental methods mostly adopted molecular markers or nuclear genome sequencing [30, 31]. Chloroplast genome studies were rarely carried out [33, 34].

The chloroplast (cp) is a core organelle in plants for photosynthesis [35]. The cp genome of angiosperms is haploid and maternal-inherited. It is shaped into a DNA circle with conserved quadripartite structure, including large single copy (LSC), small single copy (SSC) and two inverted repeat regions (IRs). Additionally, the cp genome has slow evolutionary rate, high copy numbers per cell and compact size with 120-170 kb in length [36]. Those characteristics of the cp genome, combined with the development of high-throughput technology, make sequencing of the complete cp genome an ideal tool for species identification and plant phylogenic evaluation [37–39]. To date, whole sequences of six *Trapa* chloroplast genomes have been published, comprised of five wild species (*T. natans* NC_042895, *T. quadrispinosa* MT941481, *T. kozhevnikovirum* MW027640, *T. incisa* MW543307 and *T. maximowiczii* KY705084) and one cultivated species (*T. bicornis* MT374084). However, no research involving comparative analysis was conducted for the *Trapa* cp genomes. For *Trapa*, more efforts should be made to analyze the interspecific difference and assess the phylogenic relation based on the complete cp sequences.

In this study, we sequenced the whole cp genomes of 13 wild *Trapa* species/taxa. Among them, two were sequenced for the secondarily time and the rest (11 out of 13) were sequenced for the first time. Given the unresolved taxonomic status and variable morphological characteristics of *Trapa*, the comprehensive analysis of cp genomes was only be carried out on species with detailed descriptions of taxonomic criteria and seed characteristics. Here, comprehensive analysis was first carried out within *Trapa* genus based on the 15 cp genomes data, including the 13 generated in this study and two published (*T. kozhevnikovirum* and *T. incisa*) *Trapa* species/taxa. Our specific goals were as follows: (1) to compare the chloroplast structures within *Trapa*; (2) to detect the highly variable regions as potential DNA barcoding markers for *Trapa* species identification; (3) to infer the phylogenetic relationships among *Trapa* species. This study will provide the baseline information for phylogeography within *Trapa* and facilitate the management and utilization of genetic resources of *Trapa*.

## Results

### Basic structure of chloroplast genome

**Fig. 1:**
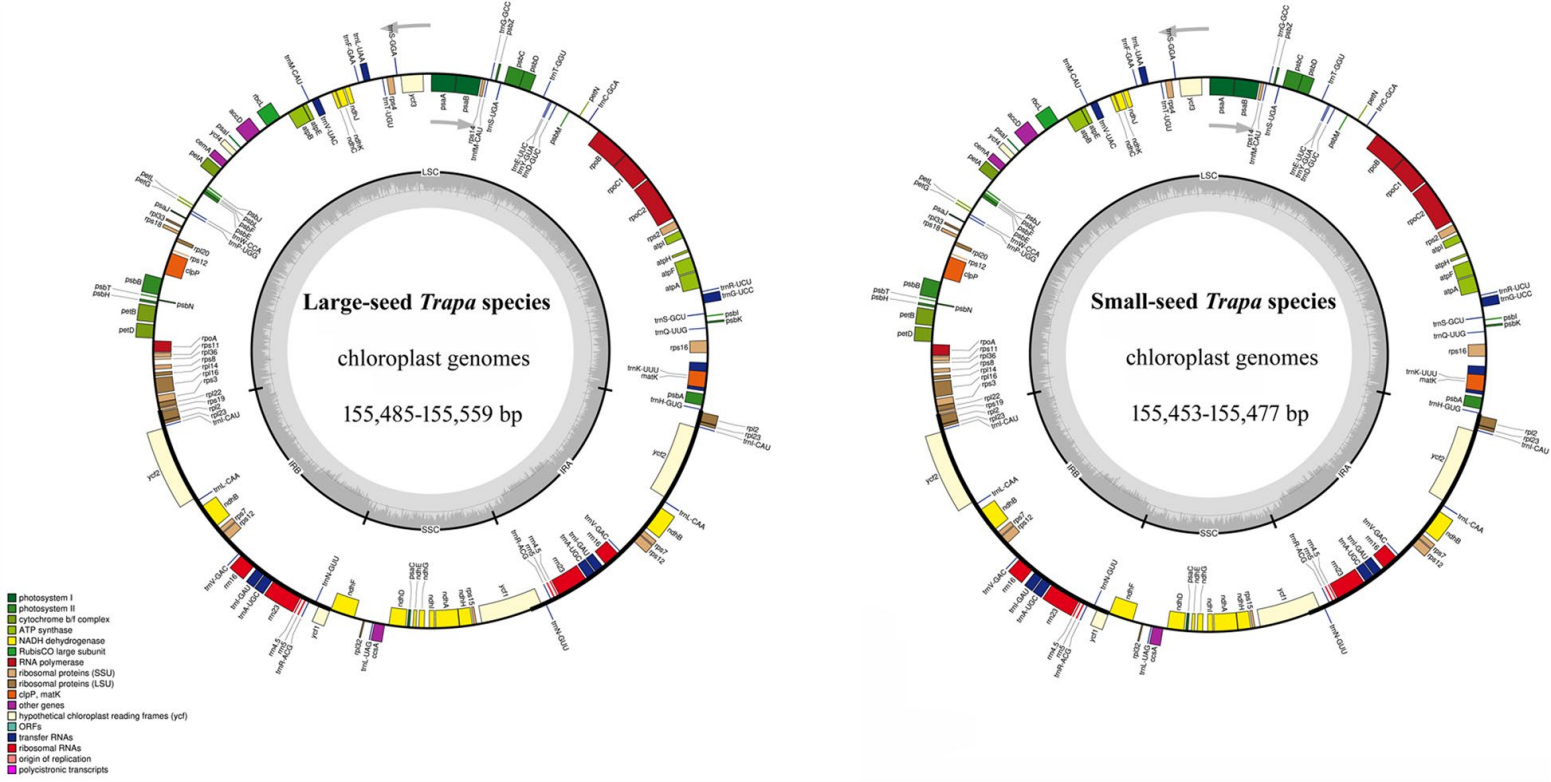
Structural map of the *Trapa* chloroplast genome. Genes drawn inside the circle are transcribed clockwise, and those outside are counterclockwise. Small single copy (SSC), large single copy (LSC), and inverted repeats (IRa, IRb) are indicated. Genes belonging to different functional groups are color-coded

The lengths of the 15 *Trapa* cp genomes varied from 155,453 to 155,559 bp. The two small-seed species showed the smallest cp genome size with 155,453 for *T. incisa* and 155,477 for *T. maximowiczii*. For the 13 *Trapa* species/taxa with large seeds, the cp genomes of the three species/taxa (*T. bispinosa*, *T. quadrispinosa* and *T. macropoda* var. *bispinosa*) showed smaller size with 155,485–155,495 bp, and the size of the others varied from 155,535 (*T. litwinowii*) to 155,559 bp (*T. mammillifera* and *T. baidangensis*) (Table 1). There were 130 genes annotated in the cp genomes of the 15 *Trapa* species/taxa, containing 85 protein-coding genes (PCGs), 37 tRNA genes and 8 rRNA genes. For the 15 *Trapa* species/taxa, among the unique genes, 44 genes were related to photosynthesis and 59 genes were associated with self-replication (Table 2); same with Lythraceae, all of the 15 cp genomes of *Trapa* species/taxa consistently showed that the gene *rpl*2 lost an intron (Fig. 1). Typical quadripartite structure was also found in *Trapa*, which consisted of a pair of IRs (26,380 – 26,388 bp) separated by the SSC (18,265–18,279 bp) and LSC regions (88,398–88,512 bp) (Table 1).

**Table 1:** Summary of complete chloroplast genomes for 15 *Trapa* species

Taxon	chLJ	xlSJ	chQJ	chSL	bdZE	hkDB	tyE	nqG	fGJ	xkGL	qqDB	jxKF	wyXBLY	SJKY	XGY
Total length(bp)	155,489	155,485	155,555	155,559	155,559	155,495	155,555	155,535	155,555	155,556	155,547	155,545	155,548	155,453	155,477
GC(%)	36.41	36.41	36.40	36.40	36.40	36.41	36.40	36.40	36.40	36.40	36.41	36.41	36.40	36.41	36.41
Length of LSC(bp)	88,444	88,440	88,504	88,508	88,508	88,450	88,504	88,485	88,504	88,505	88,512	88,494	88,498	88,398	88,428
GC of LSC(%)	34.18	34.18	34.18	34.18	34.18	34.18	34.17	34.18	34.18	34.18	34.18	34.18	34.17	34.20	34.19
Length of SSC(bp)	18,273	18,273	18,277	18,277	18,277	18,273	18,277	18,274	18,277	18,277	18,275	18,265	18,274	18,279	18,273
GC of SSC(%)	30.20	30.21	30.18	30.18	30.18	30.20	30.17	30.19	30.17	30.17	30.19	30.20	30.18	30.17	30.17
Length of IR(bp)	24,386	24,386	24,387	24,387	24,387	24,386	24,387	24,388	24,387	24,387	24,380	24,388	24,388	24,388	24,388
GC of IR(%)	42.77	42.77	42.77	42.77	42.77	42.77	42.77	42.77	42.77	42.77	42.77	42.77	42.77	42.77	42.77
Total number of genes	130	130	130	130	130	130	130	130	130	130	130	130	130	130	130
Protein-coding gene	85	85	85	85	85	85	85	85	85	85	85	85	85	85	85
tRNA	37	37	37	37	37	37	37	37	37	37	37	37	37	37	37
rRNA	8	8	8	8	8	8	8	8	8	8	8	8	8	8	8

**Table 2:** Genes in the sequenced *Trapa* chloroplast genome

Category of genes	Function of genes	Name of genes
Subunits of ATP synthase	Genes for photosynthesis	*atp A, B, E, F, H, I*
Other genes		*accD, ccsA, cemA, clpP, matK*
Subunits of NADH dehydrogenase	Genes for photosynthesis	*ndh A, B(2), C, D, E, F, G, H, I, K, J*
Subunits of photosystem I		*Psa A, B, C, I, J*
Subunits of photosystem II	Genes for photosynthesis	*psb A, B, C, E, F, G, H, I, J, K, L, M, N, T, Z*
Subunits of cytochrome		*pet A, B, D, G, L, N*
Large subunit of Rubisco	Genes for photosynthesis	*rbcL*
Large subunit of ribosome	Self-replication	*rps 2(2), 3, 4, 7(2), 8, 11, 12(2)*^*A*^*, 14, 15, 16, 18, 19*
DNA dependent RNA polymerase	Self-replication	*rpoA, B, C1, C2*
Ribosomal RNA genes	Self-replication	*rrn* 5(2)*,*4.5(2), 16(2), 23(2)
Small subunit of ribosome	Self-replication	*rpl*2(2), 14, 16, 20, 22, 23(2), 32, 33, 36
Transfer RNA genes	Self-replication	*trnA-UGC*(2)*, trnC-GCA, trnD-GUC, trnE-UUC, trnF-GAA, trnfM-CAU, trnG-GCC, trnG-UCC, trnH-GUG, trnI-CAU*(2), *trnI-GAU*(2), *trnK-UUU, trnL-CAA*(2)*, trnL-UAA, trnL-UAG, trnM-CAU, trnN-GUU*(2)*, trnP-UGG, trnQ-UUG, trnR-ACG*(2)*, trnR-UCU, trnS-GCU, trnS-GGA, trnS-UGA, trnT-GGU, trnT-UGU, trnV-GAC*(2), *trnV-UAC, trnW-CCA, trnY-GUA*
Conserved open reading frames	Genes of unknown function	*ycf1(2), 2(2), 3, 4*

(2) indicates the gene duplicated in all species

The 15 *Trapa* species/taxa showed the identical level of GC content with the total content of 36.40–36.41%. The GC content of rRNA genes reached to 55.48%, which resulted in the highest GC content in the IR regions (42.77%) compared with that of the other two regions (LSC, 34.17–34.20%; SSC, 30.17–30.21%) (Fig. 1; Table 3).

**Table 3:** Distribution of genes and intergenic regions for 15 *Trapa* species

Species	Protein Coding Genes		rRNA		tRNA		Intergenic Regions		Intron	
	Length(bp)	GC(%)	Length(bp)	GC(%)	Length(bp)	GC(%)	Length(bp)	GC(%)	Length(bp)	GC(%)
chLJ	78,672	37.28	9034	55.48	2812	53.31	46,713	30.31	18,838	36.14
xlSJ	78,672	37.28	9034	55.48	2812	53.31	46,713	30.31	18,834	36.15
chQJ	78,672	37.27	9034	55.48	2812	53.31	46,791	30.28	18,826	36.16
chSL	78,672	37.27	9034	55.48	2812	53.31	46,795	30.28	18,826	36.16
bdZE	78,672	37.27	9034	55.48	2812	53.31	46,795	30.29	18,826	36.16
hkDB	78,672	37.28	9034	55.48	2812	53.31	46,723	30.30	18,834	36.15
tyE	78,672	37.27	9034	55.48	2812	53.31	46,791	30.28	18,826	36.16
nqG	78,672	37.27	9034	55.48	2812	53.31	46,770	30.31	18,827	36.14
fGJ	78,672	37.27	9034	55.48	2812	53.31	46,791	30.28	18,826	36.16
xkGL	78,672	37.27	9034	55.48	2812	53.31	46,792	30.28	18,826	36.16
qqDB	78,672	37.27	9034	55.48	2812	53.31	46,774	30.30	18,835	36.14
jxKF	78,627	37.24	9030	55.48	2788	55.44	46,812	30.42	18,852	36.14
wyXBLY	78,498	37.27	9034	55.48	2812	53.31	46,770	30.29	18,840	36.14
SJKY	78,510	37.24	9030	55.48	2788	55.44	46,456	30.44	16,424	37.08
XGY	78,504	37.24	9030	55.48	2788	55.44	46,493	30.44	16,418	37.05

### Boundaries of IR regions and codon usage

**Fig. 2:**
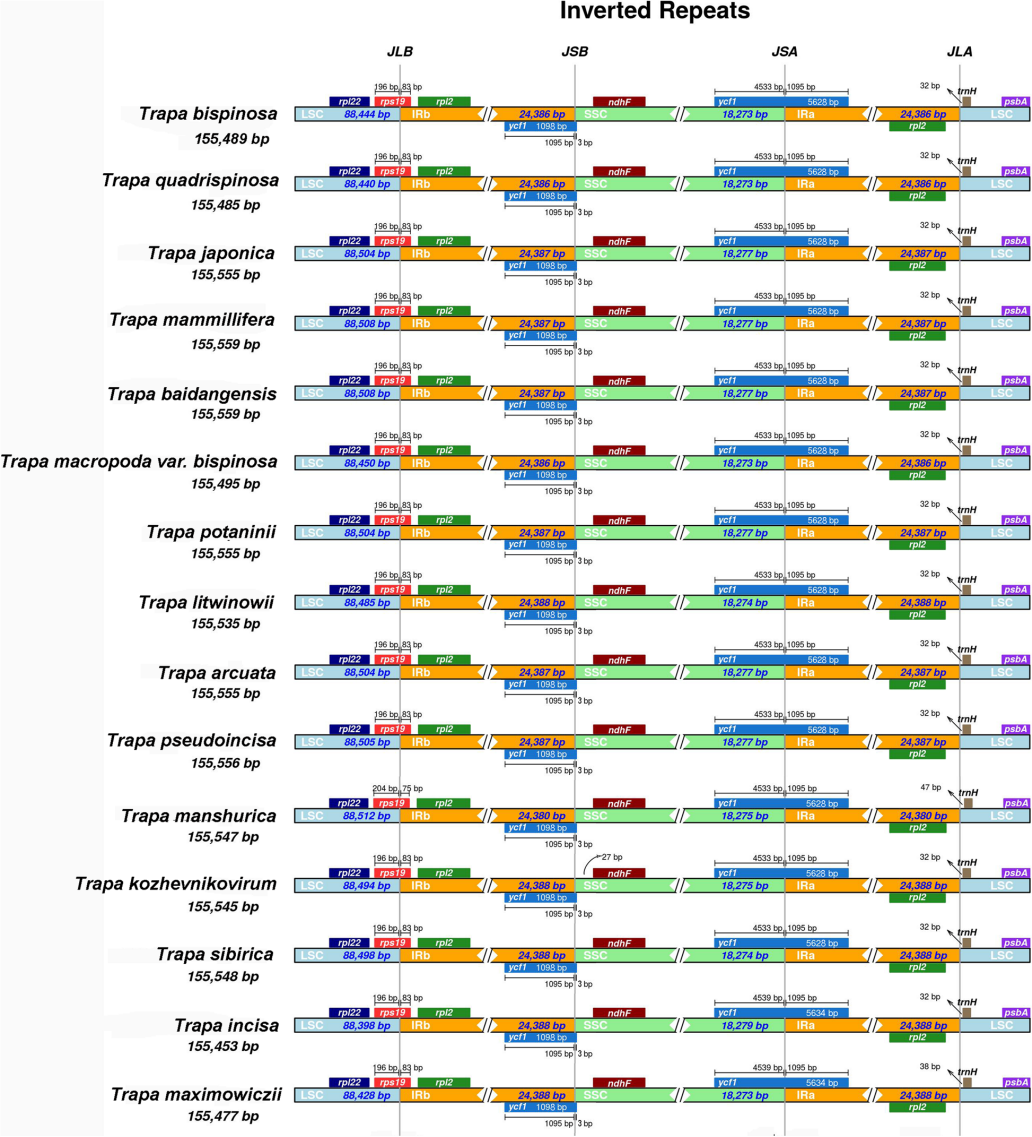
Comparison of junctions between the LSC, SSC, and IR regions among 15 species. Distance in the figure is not to scale. LSC, Large single-copy; SSC, Small single-copy; IRa and IRb, inverted repeats. JLB, junction between LSC and IRb; JSB, junction between SSC and IRb; JSA, junction between SSC and IRa; JLA junction between LSC and IRa

The cp genomes of the 15 *Trapa* plants showed several minor differences in the boundary regions although the number and order of genes were highly conserved (Fig. 2). The *rps*19 gene spanned the LSC/IRb border and extended into the IRb region with the length of 75–83 bp (except *T. manshurica* with 75 bp, the others were 83 bp). The *ycf*1 gene covered the junction of SSC/IRa showed variable sizes with 5628 or 5634 bp, which extended into IRa by 1095 bp and SSC region by 4533 (for the 13 large-seed *Trapa* taxa) or 4539 bp (for the two small-seed *Trapa* species). The gene *ycf*1 in the border of IRb/SSC showed stable length (1098 bp) extending identical distance into the IRb (1095 bp) and SSC (3 bp) for all the *Trapa* taxa. The gene *trnH* was distributed on the right side of the border of IRb/LSC, with an interval of 32–47 bp from the border to the gene.

For all the *Trapa* species/taxa, a total of 64 types of codons encoding 20 amino acids were detected. In all, 85 PCGs within *Trapa* encoded 26,160 to 26,590 codons. The codons in the 15 cp genomes of *Trapa* manifested consistent utilization mode. For example, the four codons (GAA, UUU, AUU and AAA) showed a high number of occurrences (> 1000) for all the 15 *Trapa* species/taxa; additionally, the high-frequency amino acids for all *Trapa* species/taxa were leucine (2771–2826), isoleucine (2229–2298) and serine (1992–2067), and the low-frequency amino acids were cysteine (288–298) and tryptophan (459–468).

**Fig. 3:**
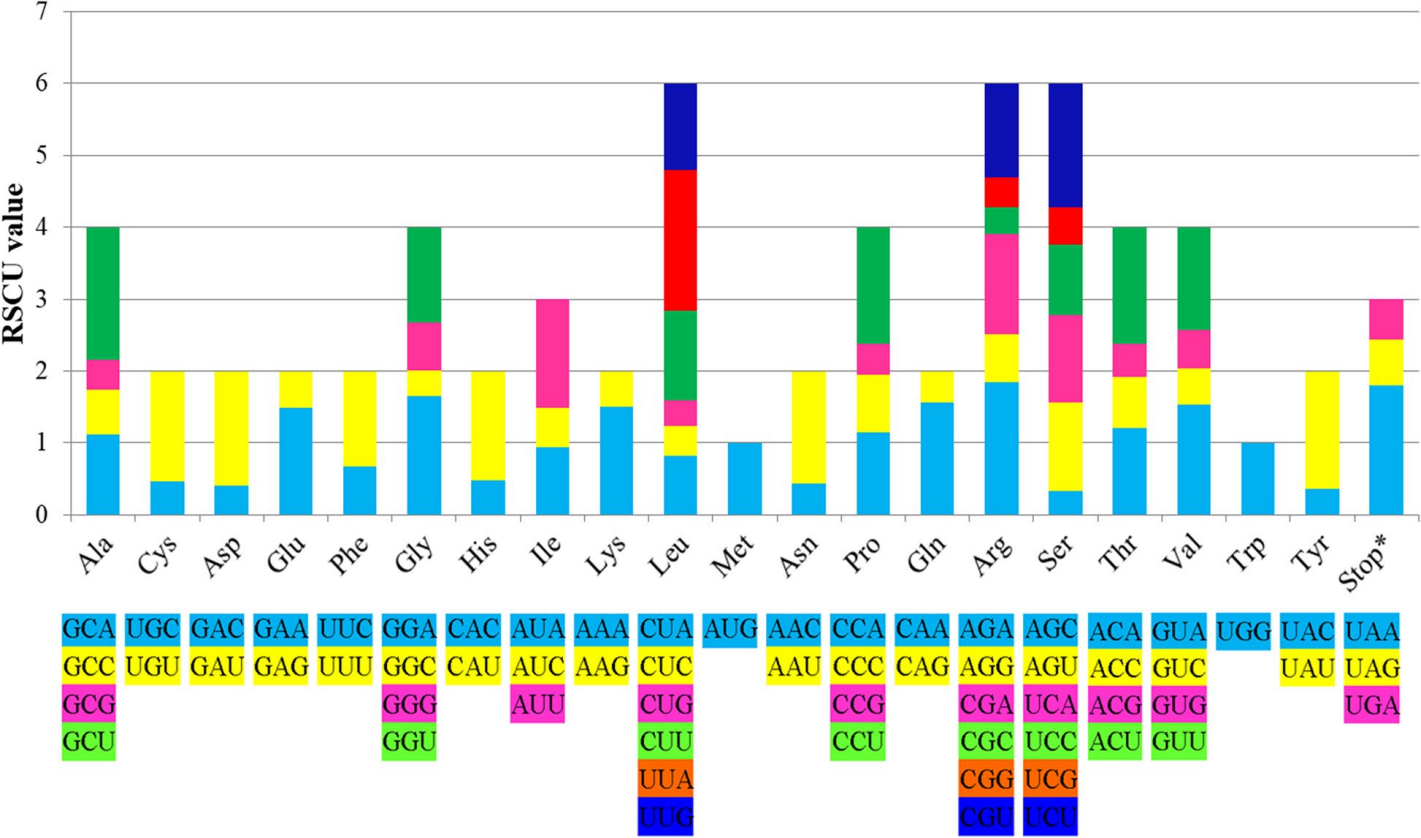
Codon contents of 20 amino acids and stop codon of coding genes of *Trapa* chloroplast genome. Color of the histogram corresponds to the color of codons

The highest and lowest relative synonymous codon usage (RSCU) values were exhibited in UUA encoding Leucine (1.96) and AGC encoding Serine (approximately 0.34), respectively (Fig. 3). The results also showed that 30 codons were used frequently with RSCU values > 1, and all of them ended with A/U.

### Hypervariable regions and comparative genomic analysis

Multiple alignments of sequences revealed a high sequence similarity across the 15 *Trapa* cp genomes, indicating that the genome structure was quite conserved both in gene identity and order (Fig. S1). Some sequence divergences were observed in the LSC and SSC regions. Notably, most of these higher variable regions were observed in conserved non-coding sequences (CNS), and a few existed in PCGs, such as *ycf*1.

**Fig. 4:**
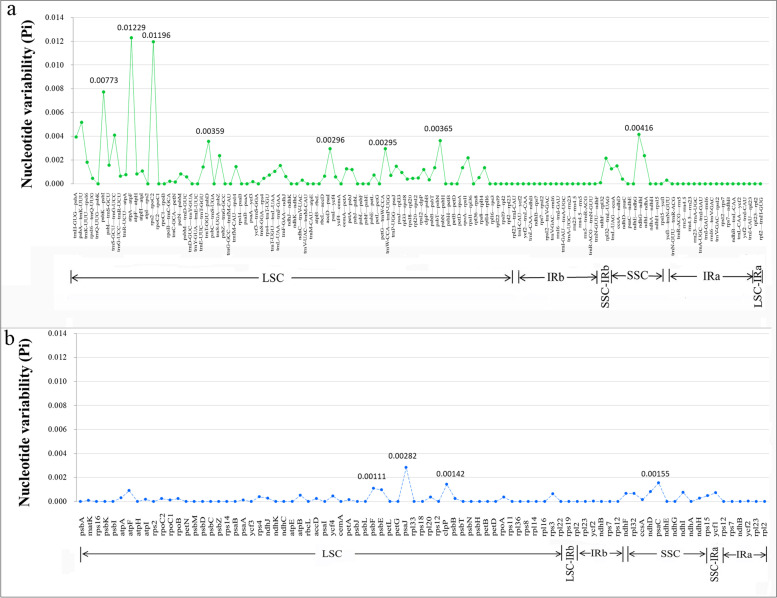
The nucleotide variability (Pi) values in the 15 *Trapa* chloroplast genomes. **a** Intergenic regions. **b** Protein-coding genes. These regions are arranged according to their location in the chloroplast genome

The intergenic regions (IGS) and introns among the 15 *Trapa* species/taxa ranged from 46,456 to 46,812 bp and from 16,418 to 18,852 bp, respectively (Table 3). The nucleotide diversity (Pi) values ranged from 0 to 0.0123 in the non-coding regions (average 0.000857) and from 0 to 0.00282 in the coding regions (average 0.000217) (Fig. 4), which indicated that the PCGs were more conserved than IGS. IGS regions of LSC possessed two divergence hotspots (*atpA—atpF* and *rps2—rpoC2*; Pi > 0.01) and two high variability (*psbA—trnK-UUU* and *psbK—psbI*; Pi > 0.005) [41] (Fig. 4).

The number of genes containing introns was 16 for all *Trapa* species. Such genes included 6 tRNA genes (*trnK-UUU*, *trnA-UGC*, *trnI-GAU*, *trnG-UCC*, *trnV-UAC* and *trnL-UAA*) and 8 PCGs (*rps16*, *rpoC1*, *atpF*, *ndhB*, *ndhA*, *petD*, *petB and rpl16*) with one intron, and two PCGs (*ycf3* and *clpP*) with two introns. For all the *Trapa* taxa, the position and length of introns in the PCGs were similar (Table 4), suggesting the high conservation of cp genomes within *Trapa*.

**Table 4:** Length of introns and complete gene of intron-contained protein-coding genes

*rps16*	*rpoC1*	*atpF*	*ndhB*	*ndhA*	*petD*	*petB*	*rpl16*	*ycf3*	*clpP*
866/1133	740/2780	766/1321	684/2217	1059/2151	742/1225	790/1438	1012/1420	756/770/2033	608/818/2017
866/1133	740/2780	766/1321	684/2217	1059/2151	742/1225	790/1438	1012/1420	756/766/2029	608/818/2017
864/1131	740/2780	767/1322	684/2217	1060/2152	742/1225	790/1438	1003/1411	756/766/2029	608/819/2018
864/1131	740/2780	767/1322	684/2217	1060/2152	742/1225	790/1438	1003/1411	756/766/2029	608/819/2018
864/1131	740/2780	767/1322	684/2217	1060/2152	742/1225	790/1438	1003/1411	756/766/2029	608/819/2018
866/1133	740/2780	766/1321	684/2217	1059/2151	742/1225	790/1438	1012/1420	756/766/2029	608/818/2017
864/1131	740/2780	767/1322	684/2217	1060/2152	742/1225	790/1438	1003/1411	756/766/2029	608/819/2018
865/1132	740/2780	758/1313	684/2217	1058/2150	742/1225	790/1438	1012/1420	757/766/2030	608/820/2019
864/1131	740/2780	767/1322	684/2217	1060/2152	742/1225	790/1438	1003/1411	756/766/2029	608/819/2018
864/1131	740/2780	767/1322	684/2217	1060/2152	742/1225	790/1438	1003/1411	756/766/2029	608/819/2018
865/1132	740/2780	766/1321	684/2217	1059/2151	742/1225	790/1438	1011/1419	757/766/2030	608/820/2019
869/1136	740/2801	767/1322	684/2163	1059/2151	742/1225	790/1438	1012/1420	757/766/2030	608/820/2019
865/1132	740/2780	768/1323	684/2217	1058/2150	742/1225	790/1438	1013/1421	757/768/2032	608/820/2019
865/1132	740/2780	768/1323	685/2218	1059/2151	751/1234	790/1438	1011/1419	761/766/2034	605/819/2015
865/1132	740/2780	768/1323	685/2218	1057/2149	751/1234	790/1438	1014/1422	758/766/2031	606/820/2017

### Long repeat and simple sequence repeats (SSRs)

A total of 595 long repeats (30–65 bp) were identified from the 15 *Trapa* taxa, consisting of 324 forward, 230 palindromic, 25 reverse and 16 complementary repeats (Fig. S2). For the *Trapa* genus, the size of the top three most frequently shown long repeats was 30 bp, 31 bp and 65 bp occurring 227, 77 and 60 times, respectively. For each species/taxon, the number of long repeats varied from 37 (*T. incisa* and *T. maximowiczii*) to 41 (*T. sibirica*); and the number of forward, palindromic, reversed and complementary repeats were 19–24, 15–16, 0–3 and 1–2, respectively (Fig. S2). Most long repeats were distributed in intergenic areas, and a few existed in shared genes, such as *ycf2*.

For each species/taxon, the number of total SSRs was from 110 (*T. quadrispinosa*) to 123 (*T. incisa* and *T. maximowiczii*). Most cp SSRs, with the proportion from 83.48% (*T. japonica*, *T. manshurica* and *T. litwinowii*) to 86.18% (*T. maximowiczii*) out of the total number of SSRs, were distributed in the LSC regions. Among these SSRs, the mononucleotide A/T repeat units occupied the highest proportion with 78.18–80.49% out of the total number of SSRs, and the second and third highest proportions were dinucleotide repeats (AT/TA and CT/TG) and tetranucleotide repeats (AAAT/AACC/AATA/AGAA/ATAG/ATGT/GGTT/TAAG/TTAA/TTTC) accounting for 9.40–10.00% and 8.13–9.09% out of the total number of SSRs, respectively (Fig. S3). The observed high AT content in cp SSRs was found in the *Trapa* genus*.*

### Phylogenetic analysis

Based on the whole cp genomes, the three phylogenetic trees, Maximum Likelihood (ML), Maximum Parsimony (MP) and Bayesian Inference (BI), showed similar topologies, and the species from the same family clustered together. The branch with the two *Lagerstroemia* species (*L. calyculata* and *L. intermedia*, Lythraceae) shown as the basal lineage. *Sonneratia alba* (Sonneratiaceae) showed to be a sister of *Trapa* species.

**Fig. 5:**
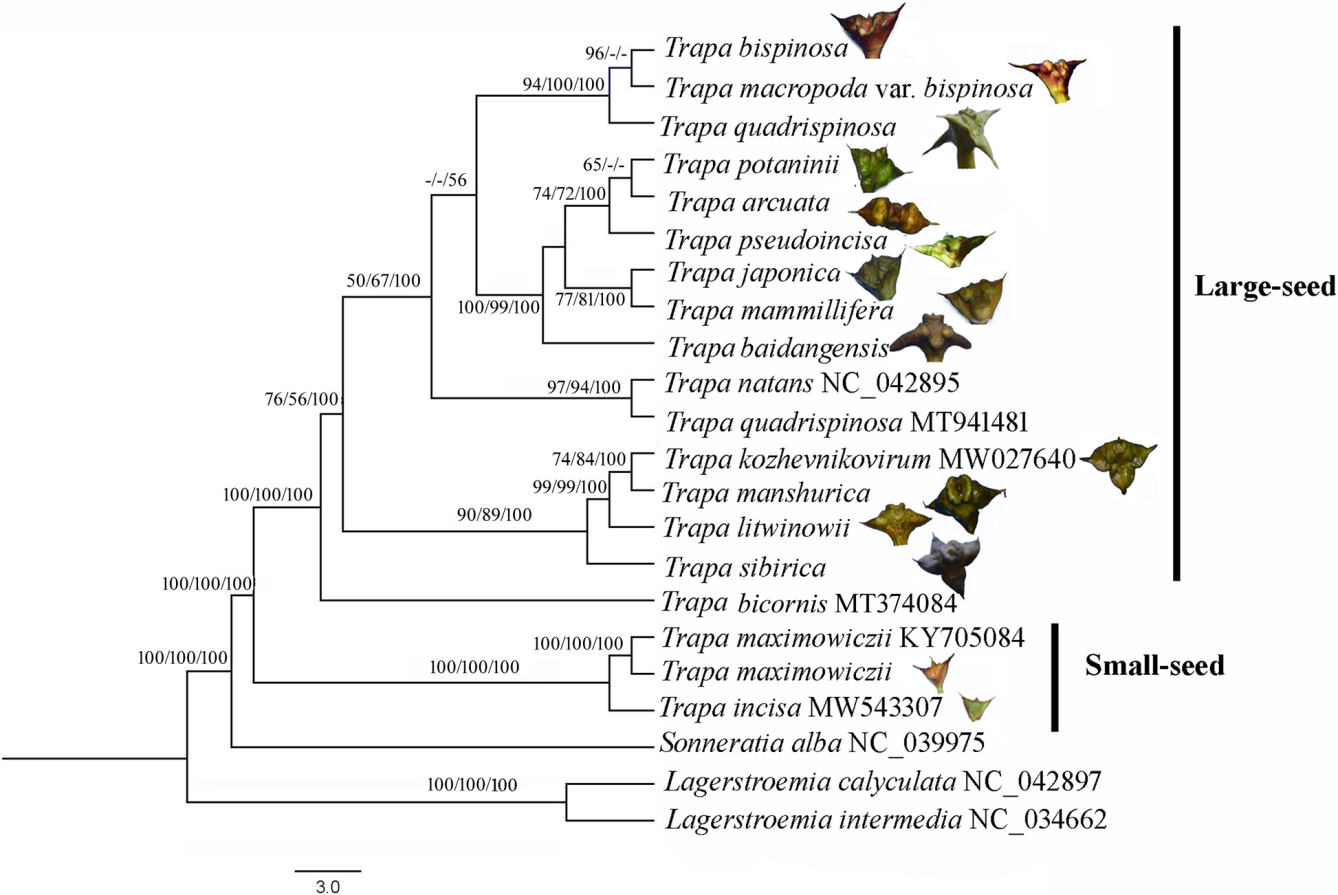
The phylogenetic tree is based on 22 complete chloroplast genome sequences using Maximum likelihood (ML), Maximum parsimony (MP) and Bayesian inference (BI) analyses. The number above the lines indicates bootstrap values (BV) for ML and MP, and posterior probabilities (PP) for BI of the phylogenetic analysis for each clade

The *Trapa* species/taxa were divided into two clusters, including small- and large-seed species/taxa (Fig. 5). The two species (*T. maximowiczii* and *T. incisa*) with small seeds were separated from the other *Trapa* taxa with high supports: bootstrap values (BV) of ML and MP trees were 94% and 100%, and posterior probabilities (PP) of BI tree was 100%. Additionally, the two small-seed species showed a sister relationship. In the cluster with large seeds, the cultivated species *T. bicornis* was the earliest-diverging species. Given that there were no seed picture and identification criteria for further species confirmation of the published species *T. quadrispinosa* (MT941481) and *T. natans* (NC_042895), it is difficult to further clarify the phylogenetic relationships of them. Among the 16 large-seed *Trapa* samples, 13 samples involved in this study were divided into three sub-clusters corresponding to their morphology: (1) the first sub-cluster included three *Trapa* species (*T. quadrispinosa*, *T. bispinosa* and *T. macropoda* var. *bispinosa*) with high BV (94% and 100%) and PP (100%); (2) the second sub-cluster contained six species/taxa (*T. japonica*, *T. mammillifera*, *T. potaninii*, *T. pseudoincisa*, *T. arcuata* and *T. baidangensis*), which showed BV of 100%, 99% and PP of 100% supports. The six species/taxa were of the group containing obvious tubercles and thick husks; (3) the third sub-cluster containing four species (*T. litwinowii*, *T. manshurica*, *T. kozhevnikovirum* and *T. sibirica*) with BV of 90%, 89% and PP of 100%. All of them have smooth and tight seed coats and were collected from the Amur River (Fig. 5).

## Discussion

### Structure and comparative analysis

The comparative analysis of cp genomes has been used extensively in many plant taxa, including plants of Lythraceae, a close relative of Trapaceae. However, it is the first time that this analysis is performed in Trapaceae. Previous studies showed that the length of land plant cp genomes ranged from 120-170 kb [36]. In the present study, the 15 *Trapa* species/taxa (155,453–155,559 bp) have an intermediate cp genome size compared to the plants from Lythraceae (152,049–160,769 bp) [42, 43]. The length of the LSC, SSC and IR regions for the 15 *Trapa* cp genomes were 88,398–88,512, 18,265–18,279 and 26,380–26,388 bp, respectively. This suggested that the difference in cp genome size of wild *Trapa* species mainly occurred in LSC region (Table 1). Most previous studies showed that the number of annotated cp genes was stable within the genus, except the two genera *Camellia* [44] and *Aquilaria* [45]. For *Trapa*, the number of genes was stable, with 130 genes including 85 PCGs, 8 rRNA genes, and 37 tRNA genes. An important evolutionary event for Lythraceae was the loss of *rpl2* intron in cp genomes, which was presumed to occur after the divergence of Lythraceae from Onagraceae [42, 43, 46, 47]. Similarly, the 15 *Trapa* species/taxa exhibited the *rpl2* intron loss, suggesting a close genetic relationship between Lythraceae and Trapaceae.

Genomes with high GC content are more stable and difficult to mutate [48]. The overall GC content in plastomes typically accounted for 30–40%, which varied greatly among different regions of cp genome and was usually higher in protein-coding regions [49]. In this study, the total GC content in *Trapa* (36.40- 36.41%) was comparable to that of Lythraceae (36.41–37.72%) [43, 47]. A high level of GC content was generally contained in rRNA genes of IR regions [43]. Similarly, for *Trapa*, due to the high GC content (55.48%) in rRNA genes, the highest GC content is found in the IR regions (42.77%) compared with that of LSC region (34.17–34.20%) and SSC region (30.17–30.21%).

The high stability of DNA sequences with high GC content resulted in a negative correlation between the GC content and the variability of cp genome sequences [43, 50]. In this study, the most variable regions for *Trapa* were located in IGS regions with the lowest GC content. Therefore, IGS can be used as DNA barcoding markers, which has been demonstrated in other species [51]. Additionally, the two divergence hotspots (*atpA*—*atpF* and *rps2*—*rpoC2*; Pi > 0.01) and the two high variability (*psbA*—*trnK-UUU* and *psbK*—*psbI*; Pi > 0.005), existed in IGS regions, could serve as potential molecular markers for further phylogenetic study (Fig. 4).

When a gene contains several internal stop codons, it tends to be a pseudogene [52, 53]. Alternatively, if the sequence is conserved over broad evolutionary distances and lacks internal stop codons, it tends to be a functional PCG [54]. A previous study about cp genomes for *Cardiocrinum* found a pseudogene *ψycf*1 located in the border of IRb/SSC [52]. On the contrary, the gene *ycf*1 in the border of IRb/SSC of 15 *Trapa* genomes contained a normal 5' initiation fragment (ATG) and just one stop codon at the border of the IRb/SSC. Thus, the *ycf*1 covered the junction of IRb/SSC is not pseudogene, and its counterpart ycf1 at the IRa/SSC border remains functional.

### Abundant long repeats and cp SSRs

For cp genomes, long repeats play an important role in the sequence rearrangement and recombination [43, 55]. In this study, 37 (*T. incisa* and *T. maximowiczii*) to 41 (*T. sibirica*) repeats were found in each species/taxon (Fig. S2). Like those of previous studies [43, 56], most repeats for *Trapa* were distributed in intergenic areas.

Due to a high polymorphism rate at the intra- and inter-species level, cp genome SSRs have been viewed as excellent molecular markers in population genetics and phylogenetic research [33, 43]. In this study, we found 110 (*T. quadrispinosa*) to 123 (*T. incisa* and *T. maximowiczii*) SSR loci. More than 80% of SSR loci were distributed in LSC regions of *Trapa* (Fig. S3), which was higher than that of the genus *Lagerstroemia* (66.55%) [10] and *Myrsinaceae* (74.37%) [57]. The mononucleotide (A/T) was of the highest proportion with 78.18–80.49% in the cp genomes of *Trapa*, which was proved in other genera [41, 58, 59]. Additionally, cp SSRs for *Trapa* also have high AT content which was positively correlated with variability of cp genome sequences [38].

### The highly conserved IR regions and codon usage

For angiosperms, the boundaries between IR regions and single-copy (SC) regions result in the difference of genome size by expansion or shrinkage [60, 63]. For *Trapa*, the IR regions had minor expansion or contraction (Fig. 2). The length of the *rps19* gene in *T. manshurica* extending into the IRb was shorter by 8 bp than that of the other species/taxa, and the interval of gene *trn*H to IRb/LSC border in *T. manshurica* was longer by 9–15 bp than that of the other species. For the two small-seed species (*T. incisa* and *T. maximowiczii*), a 6 bp sequence was inserted into the *ycf1* gene spaned SSC/IRa region (Fig. 2) compared with the gene in 13 large-seed species. Therefore, the gene *ycf1* was one of the important drivers for the expansion or contraction of the IRs in the small-seed *Trapa*.

For all the *Trapa* species/taxa, the 85 PCGs encoded 26,160 to 26,590 codons. The results were comparable to that of the genus *Lagerstroemia*, with 79 genes encoding 25,068—27,111 codons [43]. The consistent utilization mode in the 15 species suggested the high conservation of the cp genomes in *Trapa.* Like *Lagerstroemia* [43], the RSCU value of a single amino acid showed a positive correlation with the number of codons encoding it. Additionally, 30 frequently used codons ended with A/U, which might be associated with the high proportion of A/T in cp genomes [40].

### Phylogenetic analysis

Three phylogenetic trees (ML, MP and BI) consistently showed the *Sonneratia* was a sister genus of *Trapa*, which was also supported by recent studies [34, 62, 63].

The 15 *Trapa* species/taxa were divided into two clusters with high bootstrap values (BV of ML and MP trees: 100% and 100%) and posterior probabilities (PP of BI tree: 100%): the small-seed cluster and large-seed one (Fig. 5). The result indicated that the nut size of *Trapa* species was a diagnostic trait for the identification of genetic relationship within *Trapa*, which was also proved by the results from allozymic markers for Japanese *Trapa* [28]. Additionally, the results of two species with small seeds (*T. incisa* and *T. maximowiczii*) were the first split from the other *Trapa* taxa suggested that the two species were the earliest-diverging *Trapa* species, which was supported by the evidence from nuclear molecular markers [29, 30]. Although their seeds were similar in size and shape, it is the first time that the close genetic relationship between *T. incisa* and *T. maximowiczii* was shown by molecular methods. Within the large-seed group, the cultivated species *T. bicornis* diverged the earliest, which might suggest a complex origin of cultivated *Trapa* species. The remaining 13 large-seed *Trapa* taxa in this study were divided into three clusters based on their geographical origin and tubercles morphology on seeds: (1) The first cluster included *T. quadrispinosa*, *T. bispinosa* and *T. macropoda* var. *bispinosa* with high BV (94% and 100%) and PP (100%). All of them had the tight seed skin and were from the Yangtze River Basin. The intimate relationship between the former two has been proved by many studies [30, 31]. It was the first time to record the genetic data of *T. macropoda* var. *bispinosa*, and the only difference between this species and *T. bispinosa* is the larger seed bottom of this species. (2) The second cluster had six species/taxa (*T. japonica*, *T. mammillifera*, *T. potaninii*, *T. pseudoincisa*, *T. arcuata* and *T. baidangensis*) with BV of 100%, 99% and PP of 100% supports. These six *Trapa* species/taxa shared protruding tubercles on seed surfaces, and all of them were collected from the basins of the Yangtze River or Amur River. In contrast to this study, the AFLP study showed that *T. japonica* itself formed a single genetic cluster and didn’t show close relationship with other *Trapa* taxa [30]. This divergence might be attributed to different molecular markers or discordant patterns of nuclear and plastid DNA sequences. It is the first time for the four species/taxa (*T. potaninii*, *T. pseudoincisa*, *T. arcuata* and *T. baidangensis*) to be involved in a molecular study. The new species *T. baidangensis* was collected and described in this study for the first time. The seeds of the taxon have two horns and four tubercles similar to the *T. mammillifera*. The species was named *T. baidangensis* based on its collecting location. (3) The four species (*T. litwinowii*, *T. manshurica*, *T. kozhevnikovirum* and *T. sibirica*) clustered together at high supports with 90% and 89% BV for ML and MP analyses and 100% PP for BI analysis. All of them were from the Amur River, and had strong horns and tight and smooth coats on the seeds. Among them, only *T. litwinowii* has two horns on the seeds. *Trapa litwinowii*, *T. manshurica* and *T. sibirica* have large and outwardly curled seed crown, while *T. kozhevnikovirum* has a small seed crown and inconspicuous seed neck. However, the two published species, *T. quadrispinosa* (MT941481) and *T. natans* (NC_042895), clustered into a single branch. Given that most previously published *Trapa* species didn’t provide the identification criteria and seed pictures, and well-known taxonomic confusion in *Trapa*, we were not sure that the same naming was used in the previous studies.

## Conclusions

In summary, the sequences of 13 *Trapa* chloroplast genomes were acquired. Including the newly released and two previously published genomes, the comparative analyses of complete chloroplast genomes of 15 *Trapa* species/taxa were the first of their kind to be carried out. The 15 cp genomes are of the similar quadripartite structure with a high degree of the synteny in gene order, suggesting high sequence conservation. Similar to the plants of Lythraceae, the *rpl*2 intron loss was also found in all *Trapa* species/taxa, suggesting a close genetic relationship between Lythraceae and Trapaceae. A total of 130 genes were annotated in the 15 *Trapa* species. Abundant long repeats and SSRs show promise as potential molecular markers for the *Trapa* population genetics and phylogenetics. Phylogenetic analysis showed that *Trapa* species separated into two major evolutionary branches: large- and small-seed branches. The small-seed branch, including *T. incisa* and *T. maximowiczii*, were shown as basal lineage in the *Trapa* genus. The 13 large-seed *Trapa* species involved in this study were divided into three sub-clusters based on their geographical origin and tubercle morphology on seeds. This study provides novel genomic resources that should be useful for species identification and phylogeographic analysis of *Trapa*, which ultimately will contribute to the effective management and sustainable utilization of the limited conservation funding.

## Materials and methods

### Plant materials and DNA extraction

In the autumns of 2018 and 2019, 13 *Trapa* species/taxa were collected from the Yangtze River Basin and Amur River Basin. For the 13 *Trapa* species/taxa, 10 were recorded in Chinese Flora Republicae Popularis Sinicae (*T. bispinosa, T. quadrispinosa, T. japonica, T. mammillifera, T. macropoda* var. *bispinosa, T. litwinowii, T. arcuata, T. pseudoincisa, T. manshurica,* and *T. maximowiczii*) [64]; two species (*T. potaninii and T. sibirica*) were first recorded in Floral of USSR [16]. A new *Trapa* species was collected from Baidang Lake, Anhui province, China. The seeds of the species have two horns with the height from 13.4 to 18.3 mm and the width from 23.4 to 34.8 mm. The horns of the new species were wide and drooping, shaped like a pig's ears. The taxon was named *Trapa baidangensis.* The formal identification of all *Trapa* species in this study was undertook by Yuanyuan Chen who learned the *Trapa* identification following Prof. Wan Wenhao, the writer of the *Trapa* Genus of the Flora Republicae Popularis Sinicae (Wan, 2000). Because the *Trapa* species we collected from field were not protected species, no permission was required during the sampling process. All voucher specimens were deposited in the herbarium of Wuhan Botanical Garden (HIB; Table 5).

**Table 5:** The GenBank accession numbers of 15 species using in phylogenetic analysis

No	Abbr	Species	Location	Voucher No	GenBank No
1	chLJ	*Trapa bispinosa* Roxb	Changhu Lake, Hubei	yychen20180060	MW579848
2	xlSJ	*Trapa quadrispinosa* Roxb	Xiliang Lake, Hubei	yychen20180055	MW037838
3	chQJ	*Trapa japonica* Flerow	Changhu Lake, Hubei	yychen20180061	MW579849
4	chSL	*Trapa mammillifera Miki*	Changhu Lake, Hubei	yychen20180059	MW579850
5	bdZE	*Trapa baidangensis*	Baidang Lake, Anhui	yychen20180097	MW784170
6	hkDB	*Trapa macropoda* Miki *var. bispinosa* W. H. Wan	Haikou Lake, Hubei	yychen20180075	MW579851
7	tyE	*Trapa potaninii* V. Vassil	Tangyuan, Heilongjiang	yychen20180019	MW579852
8	nqG	*Trapa litwinowii* V. Vassil	Nongqiao, Heilongjiang	yychen20180034	MW579853
9	fGJ	*Trapa arcuata* S. H. Li et Y. L. Chang	856farm, Heilongjiang	yychen20180046	MW579854
10	xkGL	*Trapa pseudoincisa* Nakai	Xunke, Heilongjiang	yychen20180007	MW579855
11	qqDB	*Trapa manshurica* Fler	Qiqihaer, Heilongjiang	yychen20180001	MW579857
12	jxKF	*Trapa kozhevnikovirum* Pshennikova	Jixi, Heilongjiang	yychen20180042	MW027640
13	wyXBLY	*Trapa sibirica* Fler	Wuyun, Heilongjiang	yychen20180018	MW579856
14	SJKY	*Trapa incisa* Sieb. et Zucc	Wuhan Botanical Garden, Hubei	yychen20180066	MW543307
15	XGY	*Trapa maximowiczii* Korsch	Tangyuan, Heilongjiang	yychen20180023	MW579858

The fresh leaves were sampled and dried in silica gel immediately. Genomic DNA was extracted from the dry leaves according to the CTAB protocol [65]. The DNA concentration and quality were quantified by the NanoDrop 2000 microspectrophotometer (Thermo Fisher Scientific).

### Chloroplast genome sequencing and assembling

High quality DNA was used to build the genomic libraries. Sequencing was performed using paired end 150 bp (average short-insert about 350 bp) on Illumina NovaSeq 6000 at Beijing Novogene bio Mdt InfoTech Ltd (Beijing, China). To get the high quality clean data, Fastp [39] was run to cut and filter the raw reads with default settings. For the 13 *Trapa* species/taxa sequenced, 5.22 Gb (*T. mammillifera*) to 6.06 Gb (*T. bispinosa*) clean data were generated after removing adapters and low quality reads. De novo assembly was carried out using the assembler GetOrganelle v1.7 [66] with default settings. The software Geneious primer (Biomatters Ltd., Auckland, New Zealand) was employed to align the contigs and determine the order of the newly assembled plastomes, with *T. quadrispinosa* (MT941481) as reference. All the annotated cp sequences data reported here were deposited in GenBank with accession numbers shown in Table 5.

### Annotation and codon usage

We used the genome annotator PGA [67] and GeSeq [68] to annotate PCGs, tRNAs and rRNAs, according to the references of *T. quadrispinosa* (MT941481). Manual correction was carried out to locate the start and stop codons and the boundaries between the exons and introns. Using tRNAscan-SE v1.21, BLASTN searches were further performed to confirm the tRNA and rRNA genes [69]. The physical maps of cp genomes were generated by OGDRAW [70].

The RSCU was the ratio of the frequency of a particular codon to the expected frequency of that codon, which was obtained by DAMBE v6.04 [37]. When the value of RSCU is larger than 1, the codon is used more often than expected. Otherwise, when the RSCU value < 1, the codon is less used than expected [71].

### Comparative genomic analyses

Comparative genomic analyses were carried out among the 15 *Trapa* species/taxa, which included the 13 species/taxa newly sequenced, and two previously published ones (*T. kozhevnikovirum* and *T. incisa*) with the same research team[62, 63]. Notably, among the 15 *Trapa* species/taxa studied, *T. incisa* and *T. maximowiczii* have small size nuts (width, 9–14 mm; height, 9–12 mm), while the other 13 species/taxa are of large size nuts (width, 16–35 mm; height, 13–23 mm).The published cp genomes were downloaded from the National Center for Biotechnology Information (NCBI) organelle genome database (https://www.ncbi.nlm.nih.gov).

The mVISTA program in Shuffle-LAGAN mode was used to compare the 15 *Trapa* species/taxa complete cp genomes, with the annotation of *T. quadrispinosa* as a reference (MT941481). After manual multiple alignments using the program MUSCLE [72] in the software MEGA X [73], all regions, including coding and non-coding regions, were extracted to detect the hyper-variable sites. The nucleotide variability (Pi) was computed using DnaSP 5.10 [74].

### Analysis of repeat sequences and SSRs

Repeat sequences, including forward, palindromic, reverse and complement repeats, were detected by REPuter [75]. The parameters were set with repeat size of ≥ 30 bp and 90% or greater sequence identity (hamming distance of 3).

Simple sequence repeats (SSRs) were identified using MISA perl script [76], with the threshold number of repeats set as 10, 5, 4, 3, 3 and 3 for mono-, di-, tri-, tetra-, penta- and hexa-nucleotide SSRs, respectively.

### Phylogenetic analyses

Phylogenetic analyses were carried out based on 22 complete chloroplast genomes, including 19 *Trapa* cp genomes and three cp genomes of outgroups (*Sonneratia alba* and two Lagerstroemia species). Because of the close relationship between Trapaceae and Sonneratiaceae/Lythraceae [34], *Sonneratia alba* (Sonneratiaceae) and two *Lagerstroemia* species (*L. calyculata* and *L. intermedia*, Lythraceae) were used as outgroups. Except for the 13 *Trapa* cp genomes which were generated in this study, the other six published *Trapa* cp genomes and the three outgroup cp genomes were downloaded from Genbank.

The sequences were aligned using program Mafft 7.0 [77] with default parameters. The phylogenetic trees were constructed using three methods: (1) A Maximum Likelihood (ML) tree was performed using PhyML v.3.0 [78] with 5000 bootstrap replicates. The best-fit model of nucleotide substitution JC + I + G was obtained from software Jmodeltest 2 [79]. Previous molecular studies showed close genetic relationships between *Trapa* and *Sonneratia*/*Lagerstroemia* [33, 34]. Thus, *Sonneratia alba* (Sonneratiaceae) and two *Lagerstroemia* species (*L. calyculata* and *L. intermedia*, Lythraceae) were used as outgroups. The branch leading to two *Lagerstroemia* species was set as the root of the tree. The result was visible by the software Figtree v1.4 (https://github.com/rambaut/figtree/releases); (2) The Maximum Parsimony (MP) tree was obtained using the Subtree-Pruning-Regrafting (SPR) algorithm in the Mega X [73] with 5000 bootstrap values; (3) Bayesian Inference (BI) tree was built by the MrBayes v. 3.2.6 [80] with 2,000,000 generations and sampling every 5000 generations. The first 25% of all trees were regarded as “burn-in” and discarded, and the Bayesian posterior probabilities (PP) were calculated from the remaining trees.

## Supplementary Information

**Additional file 1: Figure S1.** Sequence alignment of whole chloroplastgenomes using the Shuffle LAGAN alignment algorithm in mVISTA. *Trapa quadrispinosa* waschosen to be the reference genome. The vertical scale indicates the percentidentity, ranging from 50 to 100%. **Figure S2.** Number of long repetitive repeats onthe complete chloroplast genome sequence of 15 *Trapa* species. (a) frequency of the repeats more than 30 bp, (b) frequencyof repeat types. chLJ, *Trapa bispinosa*;xlSJ, *Trapa quadrispinosa*; chQJ, *Trapa japonica*; chSL, *Trapa mammillifera*; bdZE, *Trapa natans *var. *baidangensis*; hkDB, *Trapamacropoda* var.* bispinosa*; tyE, *Trapa potaninii*; nqG, *Trapa litwinowii*; fGJ, *Trapa arcuata*; xkGL, *Trapa pseudoincisa*; qqDB, *Trapa manshurica*; jxKF, *Trapa kozhevnikovirum*; wyXBLY, *Trapa sibirica*; SJKY, *Trapa incisa*; XGY,* Trapa maximowiczii*. **Figure S3.** The comparison of simple sequencerepeats (SSRs) distribution in 15 chloroplast genomes. (a) frequency of commonmotifs; (b) number of different SSR types. chLJ, *Trapa bispinosa*; xlSJ, *Trapaquadrispinosa*; chQJ, *Trapa japonica*;chSL, *Trapa mammillifera*; bdZE, *Trapa natans *var. *baidangensis*; hkDB, *Trapamacropoda* var.* bispinosa*; tyE, *Trapa potaninii*; nqG, *Trapa litwinowii*; fGJ, *Trapa arcuata*; xkGL, *Trapa pseudoincisa*; qqDB, *Trapa manshurica*; jxKF, *Trapa kozhevnikovirum*; wyXBLY, *Trapa sibirica*; SJKY, *Trapa incisa*; XGY,* Trapa maximowiczii*.

## Data Availability

All the annotated cp sequences data reported here were deposited in GenBank (https://www.ncbi.nlm.nih.gov/) with accession numbers shown in Table 5. All voucher specimens were deposited in the herbarium of Wuhan Botanical Garden.

## Abbreviations

APG: Angiosperm Phylogeny Group
cp: Chloroplast
PCGs: Protein-coding genes
IR(s): Inverted repeat (regions)
IRa, IRb: Two IR regions that are identical but in opposite orientations
LSC: Large single copy; SSC: Small single copy
SSRs: Simple sequence repeats
RSCU: Relative synonymous codon usage
CNS: Conserved non-coding sequences
IGS: Intergenic regions
Pi: Nucleotide diversity
ML: Maximum-likelihood
MP: Maximum Parsimony
BI: Bayesian Inference
USSR: Union of Soviet Socialist Republics
NCBI: National Center for Biotechnology Information
