# Supplementary material for: Fifteen complete chloroplast genomes of Trapa species (Trapaceae): insight into genome structure, comparative analysis and phylogenetic relationships
Source: BMC Plant Biol. 2022 May 5;22:230. doi: 10.1186/s12870-022-03608-7 (PMC9069798; doi:10.1186/s12870-022-03608-7)
Supplement: Supplementary file 1 — Additional file 1: Figure S1. Sequence alignment of whole chloroplastgenomes using the Shuffle LAGAN alignment algorithm in mVISTA. Trapa quadrispinosa waschosen to be the reference genome. The vertical scale indicates the percentidentity, ranging from 50 to 100%. Figure S2. Number of long repetitive repeats onthe complete chloroplast genome sequence of 15 Trapa species. (a) frequency of the repeats more than 30 bp, (b) frequencyof repeat types. chLJ, Trapa bispinosa;xlSJ, Trapa quadrispinosa; chQJ, Trapa japonica; chSL, Trapa mammillifera; bdZE, Trapa natans var. baidangensis; hkDB, Trapamacropoda var. bispinosa; tyE, Trapa potaninii; nqG, Trapa litwinowii; fGJ, Trapa arcuata; xkGL, Trapa pseudoincisa; qqDB, Trapa manshurica; jxKF, Trapa kozhevnikovirum; wyXBLY, Trapa sibirica; SJKY, Trapa incisa; XGY, Trapa maximowiczii. Figure S3. The comparison of simple sequencerepeats (SSRs) distribution in 15 chloroplast genomes. (a) frequency of commonmotifs; (b) number of different SSR types. chLJ, Trapa bispinosa; xlSJ, Trapaquadrispinosa; chQJ, Trapa japonica;chSL, Trapa mammillifera; bdZE, Trapa natans var. baidangensis; hkDB, Trapamacropoda var. bispinosa; tyE, Trapa potaninii; nqG, Trapa litwinowii; fGJ, Trapa arcuata; xkGL, Trapa pseudoincisa; qqDB, Trapa manshurica; jxKF, Trapa kozhevnikovirum; wyXBLY, Trapa sibirica; SJKY, Trapa incisa; XGY, Trapa maximowiczii. [file 12870_2022_3608_MOESM1_ESM.docx]

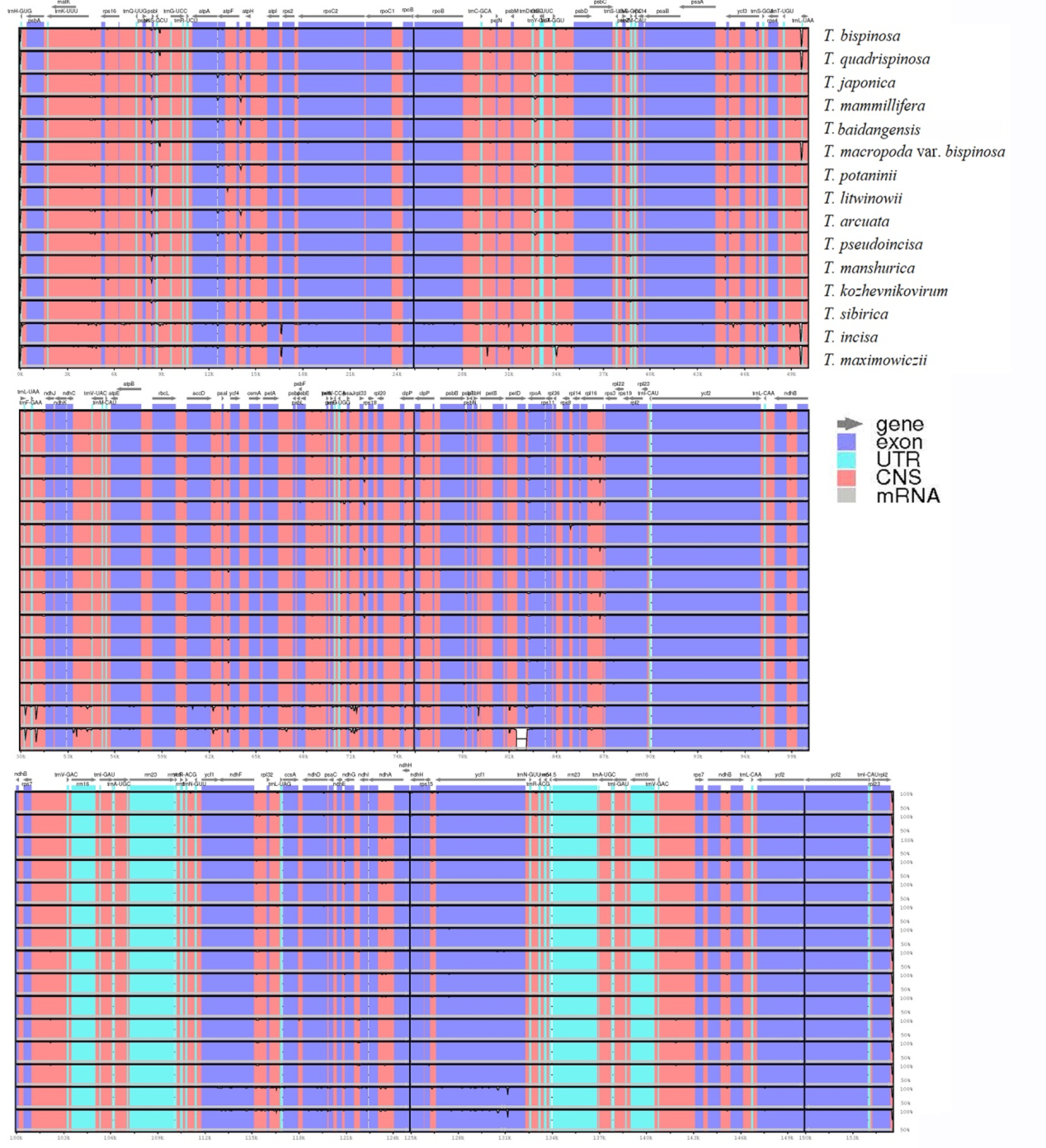


Fig. S1 Sequence alignment of whole chloroplast genomes using the Shuffle LAGAN alignment algorithm in mVISTA. *Trapa quadrispinosa* was chosen to be the reference genome. The vertical scale indicates the percent identity, ranging from 50 to 100%.


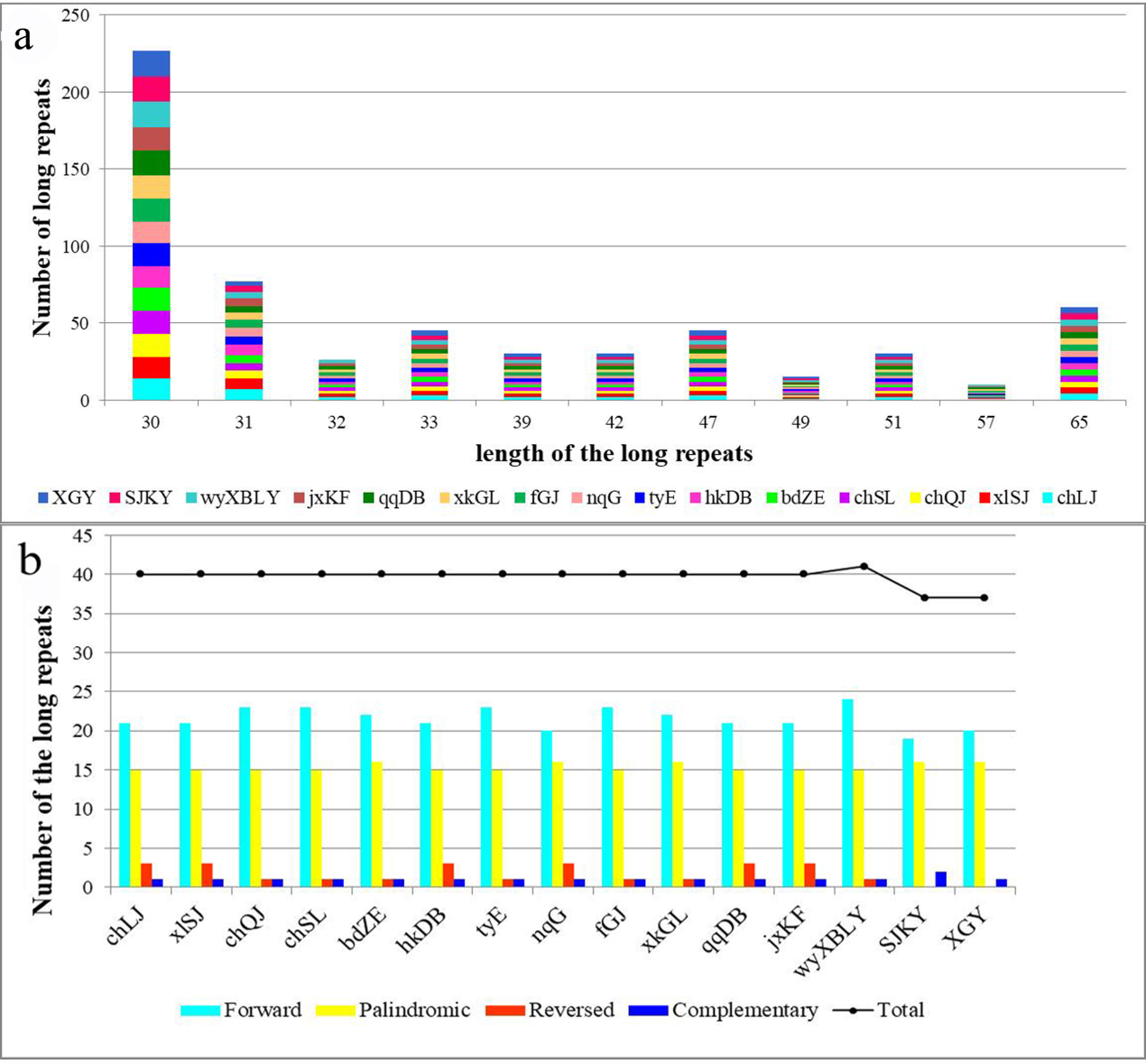


Fig. S2 Number of long repetitive repeats on the complete chloroplast genome sequence of 15 *Trapa* species. (a) frequency of the repeats more than 30 bp, (b) frequency of repeat types. chLJ, *Trapa bispinosa*; xlSJ, *Trapa quadrispinosa*; chQJ, *Trapa japonica*; chSL, *Trapa mammillifera*; bdZE, *Trapa natans* var. *baidangensis*; hkDB, *Trapa macropoda* var. *bispinosa*; tyE, *Trapa potaninii*; nqG, *Trapa litwinowii*; fGJ, *Trapa arcuata*; xkGL, *Trapa pseudoincisa*; qqDB, *Trapa manshurica*; jxKF, *Trapa kozhevnikovirum*; wyXBLY, *Trapa sibirica*; SJKY, *Trapa incisa*; XGY, *Trapa maximowiczii*.


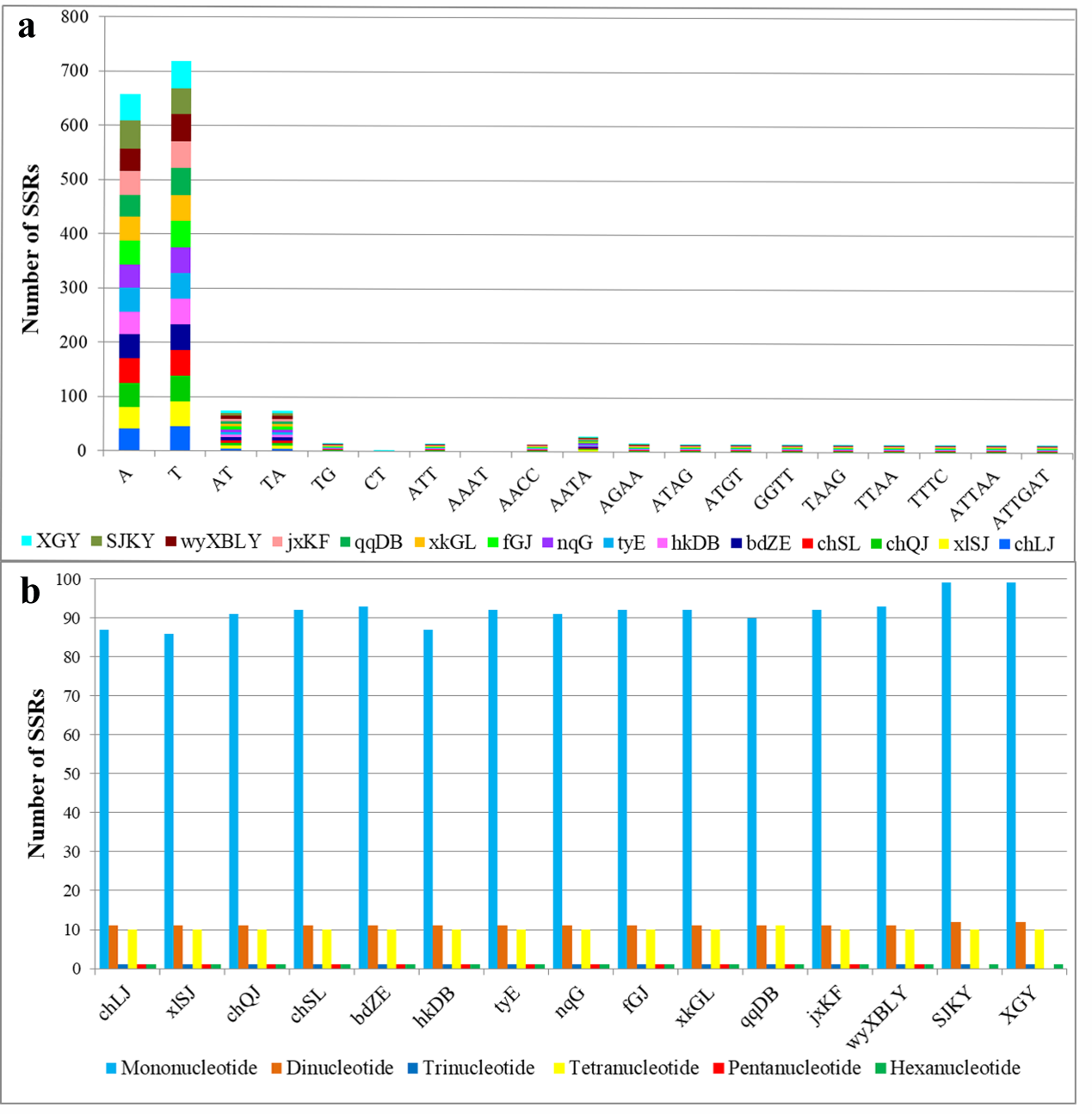


Fig. S3 The comparison of simple sequence repeats (SSRs) distribution in 15 chloroplast genomes. (a) frequency of common motifs; (b) number of different SSR types. chLJ, *Trapa bispinosa*; xlSJ, *Trapa quadrispinosa*; chQJ, *Trapa japonica*; chSL, *Trapa mammillifera*; bdZE, *Trapa natans* var. *baidangensis*; hkDB, *Trapa macropoda* var. *bispinosa*; tyE, *Trapa potaninii*; nqG, *Trapa litwinowii*; fGJ, *Trapa arcuata*; xkGL, *Trapa pseudoincisa*; qqDB, *Trapa manshurica*; jxKF, *Trapa kozhevnikovirum*; wyXBLY, *Trapa sibirica*; SJKY, *Trapa incisa*; XGY, *Trapa maximowiczii*.
